# The challenge of amyotrophic lateral sclerosis descriptive epidemiology: to estimate low incidence rates across complex phenotypes in different geographic areas

**DOI:** 10.1097/WCO.0000000000001097

**Published:** 2022-08-17

**Authors:** Giancarlo Logroscino, Daniele Urso, Rosanna Tortelli

**Affiliations:** a Center for Neurodegenerative Diseases and the Aging Brain, Department of Clinical Research in Neurology, University of Bari ’Aldo Moro’, “Pia Fondazione Cardinale G. Panico”, Tricase, Lecce; b Department of Basic Medicine, Neuroscience, and Sense Organs, University of Bari ’Aldo Moro’, Bari, Italy; c Department of Neurosciences, King's College London, Institute of Psychiatry, Psychology and Neuroscience, De Crespigny Park, London, UK; d Neuroscience and Rare Diseases Discovery and Translational Area, Roche Pharma Research and Early Development, F. Hoffmann-La Roche Ltd., Basel, Switzerland

**Keywords:** amyotrophic lateral sclerosis, epidemiology, ethnicity, global burden of disease, incidence, registry

## Abstract

Amyotrophic lateral sclerosis (ALS) is a rare progressive neurodegenerative disease of motor neurons with a fatal outcome. The rareness of the disease and the rapidly fatal course are the main challenges for the ALS epidemiological research. The understanding of ALS has clearly advanced in the recent years both in the genetics and in the leading pathways of disease determinants. Epidemiological research has played a primary role in these discoveries.

**Recent findings:**

Epidemiological studies have shown a variation of incidence, mortality and prevalence of ALS between geographical areas and different populations, supporting the notion that genetic factors, linked to populations’ ancestries, along with environmental and lifestyle factors, play a significant role in the occurrence of the disease. The burden of motor neuron diseases is increasing and currently more relevant in high-income countries but increasing at the highest rate in low and middle-income countries. The ALS phenotype is not restricted to motor functions. C9orf72 repeat expansion seems to present a recognizable phenotype characterized by earlier disease onset, the presence of cognitive and behavioural impairment.

**Summary:**

Population-based disease registries have played a major role in developing new knowledge on ALS, in characterizing genotype-phenotype correlations, in discovering new genetic modifiers and finally in planning research and health services, considering the high cost of motor neuron disease care. Epidemiological research based on multicentre international collaboration is essential to provide new data on ALS, especially in some regions of the world with poor data.

## INTRODUCTION

The number of people with neurodegenerative diseases is exponentially increasing all around the world because of the increase in the size of the world population and in the life expectancy. The main consequence of a world with more than eight billion people with a life expectancy at birth on average of more than 70 years is that in the next decades, both incidence and prevalence of neurodegenerative diseases, all age and ageing dependent, are going to increase sharply. This increase is expected to be more rapid and larger in low and medium-income countries.

**Box 1:**
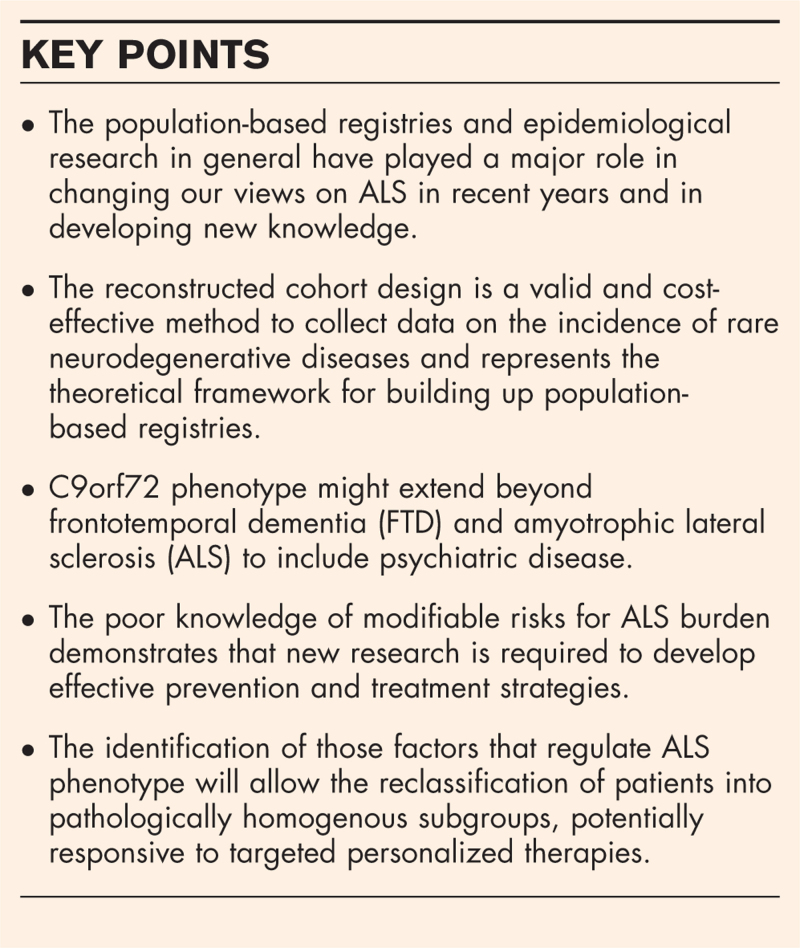
no caption available

Among the neurodegenerative diseases, ALS is considered a rare disease according of both the European Union and (prevalence < 50/100 000) [1] and the FDA (less than 200 000 affected in USA) [2]; the estimated ALS prevalence is around 10–15/100 000, at least in Europe and North America.

ALS is rapidly fatal with death intervening in 2–3 years [3]. Age is the main risk factor for ALS onset, similarly to the other more common neurodegenerative diseases such as Alzheimer's disease and Parkinson's disease. The small numbers and the course rapidly fatal course are both challenges for the epidemiological research of this devastating disease. The understanding of ALS has clearly grown in the past 30 years both in the genetics and in the leading pathways of disease determinants; in these advancements, epidemiological research has played a primary role. In this review, we are going to focus on the following topics of the ALS descriptive epidemiology, primarily developed in population settings:

(1) Population-based registries in Europe: the method challenge;
(2) ALS in the global world;
(3) ALS the complex phenotype: more than motor neuron symptoms;
(4) ALS genetics.

### Amyotrophic lateral sclerosis registries in Europe

The development of registries has been a key change of perspective for developing new knowledge on ALS. Disease registries have been identified as an essential source of information on rare diseases, in terms of clinical and epidemiologic research. They have been supported for epidemiological and public health purposes by the European Council Recommendation [4], with the primary aim to also advance the available therapies of orphan diseases. The shift in epidemiological ALS research happened in the eighties of the last century with the development of population-based registries. The first one was established in Scotland, where a prospective collaborative study was started in January 1989 within a population of about 5 millions of whom 18% were aged over 65 years [5]. Similar to the Scottish registry, ALS registries were built in the following years in Lancashire, England [6], Ireland [7] and three regions in Italy: Piemonte and Val D’Aosta (PARALS) [8], Lombardia (SLALOM) [9] and Puglia (SLAP) [10]. The population-based registries in Europe measured a relatively homogeneous incidence ranging from 1.7 to 2.3 cases per 100 000 per year, much higher than about 1/100 000, reported in most of the previous studies conducted in Europe in previous years. This common methodology across Europe was the basis for the construction of EURALS, a European consortium of all population-based registries present in the time in Europe. EURALS collected all new incident ALS cases in individuals aged 18 years and older from six population-based registries in three European countries (Ireland, UK and Italy) in the 2-year period 1998–1999, with a reference population of almost 24 million. The incidence in Europe was estimated to be 2.2/100 000 [11]. The ALS incidence had a peak in the seventies, decreased rapidly after 80 years of age and was quite homogeneous across different countries [11]. This was primarily due to the substantial improvement in the methods of data collection. One approach for studying rare neurodegenerative diseases, and in particular ALS, has been named reconstructed cohort design. As a classic cohort study design is not feasible because of the ALS rareness, we can instead use information from a complex surveillance system on incident cases during a specific time in a definite geographic region to estimate the incidence rate of a rare neurodegenerative disease. The ‘reconstruction’ of a theoretical cohort of interest represents the rationale for the assessment of the incidence in population-based registry studies [12^▪^]. This theoretical framework has been largely used in ALS and more recently in frontotemporal dementia [13,14]. One of the problems of the registries is that, even with the most accurate system of case ascertainment, there are some missing cases. Successive ALS epidemiological studies have been therefore implemented with the use of capture-recapture in population-based registers. Capture-recapture is an established and well accepted tool in studies of wildlife like those trying to estimate the number of fish of a species or with some specific characteristic in a pond. It has been proposed as a simple and economic method to conduct studies on frequency of rare disease and monitor prevalence and incidence over time [15,16].

In the registry of Limousin region (FRALIM) in Southern France, an area with less than one million of inhabitants, the use of capture recapture method showed that the number of missing cases was as high as 34% [15]. The capture recapture was obtained using three different independent sources of data: a computerized database of the Neurology Department of the University Hospital of Limoges; the neurologists of the Limousin region and neighbouring provinces; and the hospitals of the Limousin region and neighbouring provinces. In this register, the exhaustiveness has been improved and in a successive study in the same area was estimated as particularly high, at 98.4% with an estimated incidence of 3.2/100 000 [17]. The ALS register in the Netherlands was implemented with a similar methodology and in the years 2006--2009, collected 1217 incident cases with an estimated incidence, after the use of capture-recapture method, of 2.77 per 100 000 person-years [18]. A similar incidence was found in South Germany in Swabia, a region of eight million inhabitants, 2.5 and rising to 3.1 per 100 000 person-years after capture-recapture with about 18% of missing cases [19]. These studies show that even with registries well organized and with important resources, the missing cases, calculated with the capture-recapture method, are between 15 and 35%.

### Amyotrophic lateral sclerosis in the global world

The epidemiological studies have reported consistently a heterogeneity of the risk and prevalence of ALS in different world regions and have begun to question the origin of this heterogeneity [20]. A recent systematic review and meta-analysis of population-based data [21] from 45 studies in 11 subcontinents was used to assess the worldwide ALS incidence and to test the possible heterogeneity across different geographic areas. A total of 13 146 ALS cases and 825 million person-years of follow-up (PYFU) were analysed. The overall pooled worldwide crude ALS incidence was at 1.75. The highest incidence was found in Northern Europe (1.89), the lowest in East and South Asia, respectively, 0.83 and 0.73. In contrast to the extreme variation in the whole world, the incidence is relatively homogeneous in studies from North America, New Zealand and Europe. Outside of ALS clusters reported in the past like in Guam or Kiji peninsula [22,23], the highest reported ALS incidence so far has been recently reported from Faroe island [24^▪^], a small archipelago of 18 islands with a population of about 50 000 people: 4.9/100 000 in the period 2010–2020 raising from 2.6 in the period 1987–2000. This almost 70% increase has been attributed to possible environmental factors as seafood, including whale meat and blubber, components of the traditional Faroese diet. This food is more likely to be contaminated in recent years with persistent pollutants such as methylmercury and polychlorinated biphenyls (PCBs). The most comprehensive estimates of ALS frequencies are based on the analyses of Global Burden of Diseases (GBD) the project at Washington University, USA, that aims to produce estimates of incidence, prevalence and disability-adjusted life-years (DALYs) for all diseases in all countries of the world [25^▪▪^]. The study is the only one that counted all motor neuron diseases, including amyotrophic lateral sclerosis (ALS), spinal muscular atrophy, hereditary spastic paraplegia, primary lateral sclerosis, progressive muscular atrophy and pseudobulbar palsy. In 2016, globally, 330 918 [95% uncertainty interval (UI) 299 522–367 254) individuals had a motor neuron disease. Motor neuron diseases have caused more than 900 000 DALYs and almost 35 000 deaths, with a substantial impact on disability and mortality. Incidence and prevalence of motor neuron diseases is characterized by geographical heterogeneity but is not explained by any of the 84 risk factors examined in GBD. These findings suggest that other unknown risk factors might play a causal role. Of interest the possible role of the Sociodemographic Index (SDI), which is a summary measure that identifies where countries or other geographic areas sit on the spectrum of economic development. In the GBD study, there is a correlation between SDI and incidence and prevalence of ALS, with the exception of low ALS risk in countries with high SDI in East Asia. These specific results may be at least partially explained by a possible role of ethnicities and ancestries in ALS causation. This hypothesis was first proposed in a population-based study on mortality in Cuba where mixed ancestry had shown to be protective in the sporadic form of the disease with rates that were lower (0.55/100 000) in the mixed population compared with black or white people (0.93 and 0.87/100 000) [26]. This hypothesis is going to be tested in LAENALS, a study on epidemiology of ALS, in countries with mixed ethnicities in Latin America [27].

### Amyotrophic lateral sclerosis, the complex phenotype: more than a motor neuron disease

ALS was traditionally considered to spare cognitive functions and early reports of cognitive and behavioural deficits in motor neuron diseases might have been overlooked initially [28]. Although the co-occurrence of motor neuron symptoms and cognitive impairment has been reported throughout the early parts of the 20th century, the presence of cognitive impairment in ALS has not been fully recognized until the final decade of the 20th century [29]. In the last three decades, more specific associations between ALS and FTD have been identified [30]. Pathological and genetic findings [31,32] have recently reinforced the overlap between ALS and FTD and the concept of a frontotemporal dementia (FTD)-motor neuron disease continuum has clearly emerged [28,33,34]. Cognitive impairment in ALS can be very heterogeneous and is characterized by personality change, irritability, obsessions, poor insight and pervasive deficits in frontal executive tests [28]. This presentation is consistent with the changes to the character, social conduct and executive function in FTD.

In the last decade, four population-based studies carried out in Ireland and Italy have characterized the presence of cognitive dysfunction and FTD-motor neuron disease in patients with ALS (Table 1). About 10–20% of patients with motor neuron disease met criteria for a diagnosis of FTD (ALS-FTD) at baseline [35^▪▪^,^36^–^38^], but cognitive impairment without dementia might be detected in a higher proportion of patients. In fact, executive impairment has been found in about 20–25% of ALS patients, while nonexecutive cognitive impairment is present in about 2–10% of patients [35^▪▪^,^36^,^37^]. Isolated behavioural impairment is noted in about 6–13% of ALS cases [35^▪▪^,^36^,^37^], and depending on the population and the extent of cognitive testing performed, most studies have suggested that up to 50% of patients have cognitive abnormalities [35^▪▪^,^36^,^37^]. Population-based studies have also long corroborated the relation between psychotic events and ALS (Table 1). In particular, an increased risk of hospitalization for schizophrenia could be observed in the 5 years preceding ALS, with higher statistical significance especially 1 year before the onset of motor symptoms [39,40]. This close relationship between psychotic features and motor symptoms in ALS may underlie the prodromal nature of these extra-motor symptoms in the framework of ALS pathogenesis [39]. Interestingly, a Swedish register-based nested case--control study showed that family members of ALS patients, especially children, had an increased risk for manifesting psychiatric disturbances both before and after their relative's diagnosis [40]. Similarly, aggregation studies suggested neuropsychiatric illnesses and ALS clusters in families. Two Irish population-based cohort studies showed that the relative risk of developing a neuropsychiatric condition, such as schizophrenia, psychosis, obsessive-compulsive disorder, autism and alcoholism, was significantly higher in first or second-degree relatives of ALS patients [41,42]. Whether this can be explained by genetic pleiotropy of few variants into several kindreds or by a shared polygenic risk between psychiatric diseases and ALS spectrum remains to be determined [42]. However, *C9orf72* hexanucleotide repeat expansion, which can cause familial FTD, ALS and mixed phenotypes, may contribute to this association. In fact, symptomatic *C9orf72* expansion carriers display higher rates of psychotic and other psychiatric symptoms than noncarriers [43]. A recent study looking at kindreds of patients with both ALS and FTD showed that the risk of psychiatric illnesses (schizophrenia, late-onset psychosis unrelated to schizophrenia, suicide and autism spectrum disorders) was significantly greater in kindreds of C9orf72 expansion carriers than in kindreds of noncarriers [44].

**Table 1:** Population-based studies of psychiatric and cognitive features of amyotrophic lateral sclerosis

Author (year)	Domain	Country	Study design, *n*	Prevalence and findings
Turner *et al.* (2016) [39]	Psychiatric disorders	UK	Record-linkage study	• Increased risk of hospitalization for schizophrenia in the year preceding ALS (rate ratio 2.95, 95% confidence interval 2.13–4.00). • Increased risk of hospitalization for depression in the 5-year preceding ALS (rate ratio 1.50, 95% confidence interval 1.24–1.81).
Longinetti *et al.* (2017) [40]	Psychiatric disorders	Sweden	Register-based nested case--control study, *n* = 3648 ALS and *n* = 36 480 healthy controls	• Individuals with previous neurodegenerative or psychiatric diseases had a 49% increased risk of ALS (odds ratio 1.49, 95% confidence interval 1.35–1.66) • Patients with ALS had increased risks of other neurodegenerative or psychiatric diseases after diagnosis (hazard ratio 2.90, 95% confidence interval 2.46–3.43)
Phukan *et al.* 2011 [38]	Cognitive impairment	Ireland	Population-based cross-sectional study, *n* = 160 ALS and *n* = 110 healthy controls	• 13.8% of ALS patients had FTD • 34.1% of ALS patients had cognitive impairment (executive) • 14% of ALS patients had nonexecutive cognitive impairment • 46.9.1% of ALS patients had no cognitive impairment
Montuschi *et al.* 2013 [36]	Cognitive impairment	Italy	Population-based cross-sectional study, *n* = 183 ALS and *n* = 127 healthy controls	• 12.6% of ALS patients had FTD • 19.7% of ALS patients had cognitive impairment (executive) • 5.5% of ALS patients had nonexecutive cognitive impairment • 6% of ALS patients had behavioural impairment • 6% of ALS patients had nonclassifiable cognitive impairment • 49.7% of ALS patients had no cognitive impairment
Elamin *et al*. 2013 [37]	Cognitive impairment	Ireland	Population-based longitudinal study, *n* = 186 ALS	• 11.8% of ALS patients had FTD • 25.2% of ALS patients had cognitive impairment (executive) • 12.3% of ALS patients had nonexecutive cognitive impairment • 50.5% of ALS patients had no cognitive impairment
Byrne *et al.* 2012 [55]	Cognitive impairment	Ireland	Population-based longitudinal study, *n* = 191 ALS	• ALS patients with C9orf72 repeat expansion had: • Lower age at disease onset • More co-morbid FTD • Distinct neuroimaging changes at cortical level • Shorter survival
Chiò *et al.* (2019) [35^▪▪^]	Cognitive impairment	Italy	Population-based cross-sectional study, *n* = 797 ALS	• 20.5% of ALS patients had FTD • 4.8% of ALS patients had cognitive and behavioural impairment • 16.6% of ALS patients had cognitive impairment • 2.0% of ALS patients had nonexecutive cognitive impairment • 7.9% of ALS patients had behavioural impairment • 48.2% of ALS patients had no cognitive impairment

### Genetics of amyotrophic lateral sclerosis: lessons from population-based cohorts

The knowledge of the genetic landscape for ALS has progressed a lot over the past 30 years since the discovery of pathogenic mutations in SOD1 in 1993 [45]. Nowadays, in association with the disease, researchers described more than 40 genes, and the genetic cause of approximately two-thirds of familial cases (fALS) has now been elucidated [46,47]. Population-based registers have played a major role in delineating mutation rates in specific populations, in better characterizing genotype-phenotype correlations and in discovering new genetic modifiers. They are able to capture all ALS cases, independently of age, sex, socioeconomic status, disease severity, and can thus provide a complete information regarding epidemiology and disease characteristics, including genetics [48].

Very recently, the Piedmont and Valle d’Aosta Registry for ALS (PARALS), established in 1995 to study the epidemiology and characteristics of the disease in Northern Italy, provided valuable insights into the precise frequency and burden of rare genetic variants in 40 known ALS gene in that area [49]. They performed whole genome sequencing in 959 Italian patients with ALS and 677 healthy controls from the same geographical area, and they found potential disease-causing variants in 11.9% of the ALS cohort, with the C9orf72 expansion being the most frequent mutation both in sporadic and familial cases. They also found rare variants in the SOD1 gene having the strongest association with the disease risk, followed by TARDBP rare variants [49]. Furthermore, they reported rare variants in the *NEFH* gene lowering the risk of the disease [49] and overall confirmed the contribution of rare genetic variants to the disease risk. The PARALS cohort was also important for estimating the frequency of familial ALS and SOD1 mutations in Northern Italy, which turned out to be overestimated not only in clinical-based cohorts, but also of others ALS-related genes, with the risk of harbouring a causing mutation being driven by a positive family history for ALS and/or FTD, presence of comorbid FTD and younger age of onset of the disease [50]. Table 2 summarizes the frequency of the mutations in the four ALS principal Mendelian genes, reported by the three major European population-based registries for ALS: Piedmont and Valle d’Aosta, Ireland and The Netherlands.

**Table 2:** Frequency of mutations in ALS major genes, reported by three population-based cohorts in Europe

Regions	Population	C9orf72	SOD1	TARDBP	FUS/TLS
Piemonte and Valle d’Aosta, Italy	4 332 842	6.5% (70% with a family history)^a^	13.6% (fALS)^b^ 0.7% (sALS)^b^ 2.1% (fALS and sALS)^a^	1.5% (fALS and sALS)^a^	0.002% (fALS)
Ireland	3 626 087	41% (fALS)^c^ 5% (sALS)^c^	0% (fALS) ^d^ 0% (sALS) ^d^	0% (fALS) ^d^ 0.45% (sALS)^d^	0% (fALS)^d^ 0.45% (sALS)^d^
Netherlands	16 455 911	36% (fALS)^e^ 6.1% (sALS)^e^	1.0% (fALS)^e^ 0.4% (sALS)^e^	8.2% (fALS)^e^ 0.4% (sALS)^e^	6.2% (fALS)^e^ 0.3% (sALS)^e^

a Adapted from [50]. Chiò A, Calvo A, Mazzini L, *et al*. Extensive genetics of ALS: a populationbased study in Italy. Neurology 2012; 79:1983–1989.

b Chiò A, Traynor BJ, Lombardo F, *et al.* Prevalence of SOD1 mutations in the Italian ALS population. *Neurology.* 2008;70:533-537.

c Byrne S, Elamin M, Bede P, *et al.* Cognitive and clinical characteristics of patients with amyotrophic lateral sclerosis carrying a C9orf72 repeat expansion: a population-based cohort study. *Lancet Neurol.* 2012;11:232-240.

d Kenna KP, McLaughlin RL, Byrne S, *et al.* Delineating the genetic heterogeneity of ALS using targeted high-throughput sequencing. *J Med Genet.* 2013;50:776-783.

e van Blitterswijk M, van Es MA, Hennekam EA, *et al.* Evidence for an oligogenic basis of amyotrophic lateral sclerosis. *Hum Mol Genet.* 2012;21:3776-3784.

The Irish population-based register, through a very detailed analysis of family history and genotyping of patients for more than 20 years, provided important clues regarding the true proportion of fALS, closer to 16–20% of all ALS cases [41]. Furthermore, it gave a major contribution in delineating the genetic heterogeneity of ALS, considering population structure and how the frequency and the importance of the major ALS genes can vary based on population ethnicity and ancestry [51]. Of note, the frequency of C9orf72 expansion is high in European populations and low in Asian cohorts [52], or SOD1 mutations account for 13% of fALS in Italy, but are not present in Ireland, and very rare in The Netherlands [51].

Population-based studies also played an important role in investigating the genotype-phenotype relationship in ALS. Chiò *et al*. demonstrated that C9orf72 repeat expansions have a primary role in increasing the risk of cognitive impairment in patients with ALS, and only in the presence of the C9orf72 repeat expansion, the APOE ε2 allele, to a lesser extent, also increases the risk of FTD in this subpopulation [53,54^▪▪^]. Furthermore, the Irish registry has been one of the first populations for which the genotype-phenotype correlation of C9orf72 hexanucleotide expansion has been extensively investigated both in fALS and in sporadic ALS. Byrne *et al*. [55] reported that patients with ALS and the C9orf72 repeat expansion seem to present a recognizable phenotype characterized by earlier disease onset, the presence of cognitive and behavioural impairment, specific neuroimaging changes, a family history of neurodegeneration with autosomal dominant inheritance and reduced survival. The Dutch Register recently reported that the effect of lifestyle on the risk of developing ALS depends on the C9orf72 genotype [56^▪^].

Another contribution of ALS population-based registries was towards a better understanding of the heritability of ALS. A twin study from the English register estimated the heritability of ALS to be quite high as 0.61 [57]. Recently, the Irish register, using a prospective population-based parent-offspring heritability study conducted for 10 years, showed that the lifetime risk of developing ALS in first-degree relatives of individuals with ALS was increased compared with the general population, and the mean lifetime heritability of ALS was 52.3% for the overall study cohort and higher in mother-daughter pairings (66.2%) [58].

## CONCLUSION

The GBD data show that the burden of motor neuron diseases is increasing and currently more relevant in high-income countries but increasing at the highest rate in low and middle-income countries. This heavy burden is increasing primarily because of population ageing. The ALS phenotype is not restricted to motor functions. The role of behavioural and especially cognitive symptoms and signs should also be strongly considered in new classification systems [59]. The genetics and the consequent population specific ALS risk are highly variable and determined by the ethnic composition. This is clearly shown studying the changes of prevalence of C9ORF72 across Europe [60]. Geographic ALS heterogeneity is important to determine causal risk factors; we need to have more research, population-specific, as the role of diet and possible pollutants in the Faroe island show. The population-based registries and epidemiological research in general have played a major role in changing our views on ALS in recent years and inquiries on causes of ALS. Recent findings from epidemiological ALS studies are key to plan appropriate health service planning considering the high cost of motor neuron disease care. GBD data show that a global strategy is really needed, considering that a cure for motor neuron disease is probably not far away as in the past [61,62].

## Acknowledgements

*The authors thank Marco Musio, Center for neurodegenerative diseases and the Aging Brain, for the technical assistance in the preparation of this manuscript.*

### Financial support and sponsorship

*This work has been supported with the funding of Regione Puglia and CNR for Tecnopolo per la Medicina di Precisione. D.G.R. no. 2117 of 21.11.2018 (B84I18000540002).*

### Conflicts of interest

*R.T. is a full employee of F. Hoffmann-La Roche Ltd.*

## REFERENCES AND RECOMMENDED READING

Papers of particular interest, published within the annual period of review, have been highlighted as:

▪ of special interest
▪▪ of outstanding interest
